# The *Plasmodium falciparum*, Nima-related kinase Pfnek-4: a marker for asexual parasites committed to sexual differentiation

**DOI:** 10.1186/1475-2875-11-250

**Published:** 2012-07-31

**Authors:** Luc Reininger, Miguel Garcia, Andrew Tomlins, Sylke Müller, Christian Doerig

**Affiliations:** 1 CNRS USR3151, Station Biologique, Place Georges Teissier, 29680, Roscoff, France; 2 Wellcome Trust Centre for Molecular Parasitology, Institute of Infection, Immunity & Inflammation, College of Medical, Veterinary and Life Sciences, University of Glasgow, 120 University Place, Glasgow, G12 8TA, UK; 3 INSERM U609, INSERM-EPFL Joint Laboratory, GHI-SV-EPFL, Station 19, Lausanne, CH-1015, Switzerland; 4 FCCF-SV-EPFL, Station 15, Lausanne, CH-1015, Switzerland; 5 Department of Microbiology, Monash University, Wellington Road, Clayton, VIC, 3800, Australia

**Keywords:** Gametocytes, NIMA, Plasmodium falciparum, Ser/Thr protein kinase

## Abstract

**Background:**

Malaria parasites undergo, in the vertebrate host, a developmental switch from asexual replication to sexual differentiation leading to the formation of gametocytes, the only form able to survive in the mosquito vector. Regulation of the onset of the sexual phase remains largely unknown and represents an important gap in the understanding of the parasite’s complex biology.

**Methods:**

The expression and function of the Nima-related kinase Pfnek-4 during the early sexual development of the human malaria parasite *Plasmodium falciparum* were investigated, using three types of transgenic *Plasmodium falciparum* 3D7 lines: (i) episomally expressing a Pfnek-4-GFP fusion protein under the control of its cognate *pfnek-4* promoter; (ii) episomally expressing negative or positive selectable markers, yeast *cytosine deaminase*-*uridyl phosphoribosyl transferase*, or human *dihydrofolate reductase*, under the control of the *pfnek-4* promoter; and (iii) lacking a functional *pfnek-4* gene. Parasite transfectants were analysed by fluorescence microscopy and flow cytometry. *In vitro* growth rate and gametocyte formation were determined by Giemsa-stained blood smears.

**Results:**

The Pfnek-4-GFP protein was found to be expressed in stage II to V gametocytes and, unexpectedly, in a subset of asexual-stage parasites undergoing schizogony. Culture conditions stimulating gametocyte formation resulted in significant increase of this schizont subpopulation. Moreover, sorted asexual parasites expressing the Pfnek-4-GFP protein displayed elevated gametocyte formation when returned to *in vitro* culture in presence of fresh red blood cells, when compared to GFP^-^ parasites from the same initial population. Negative selection of asexual parasites expressing *pfnek-4* showed a marginal reduction in growth rate, whereas positive selection caused a marked reduction in parasitaemia, but was not sufficient to completely abolish proliferation. *Pfnek-4*^*-*^ clones are not affected in their asexual growth and produced normal numbers of stage V gametocytes.

**Conclusions:**

The results indicate that Pfnek-4 is not strictly gametocyte-specific, and is expressed in a small subset of asexual parasites displaying high rate conversion to sexual development. Pfnek-4 is not required for erythrocytic schizogony and gametocytogenesis. This is the first study to report the use of a molecular marker for the sorting of sexually-committed schizont stage *P. falciparum* parasites, which opens the way to molecular characterization of this pre-differentiated subpopulation.

## Background

The life cycle of *Plasmodium falciparum* parasites includes multiple rounds of asexual replication in human host erythrocytes. A small subset of parasites, upon invasion of new red blood cells, do not enter schizogony, but develop into cell cycle-arrested gametocytes [1]. Gametocytes are the only forms able to survive in the mosquito vector, and their formation is therefore essential for malaria transmission. How malaria parasites regulate the switch from erythrocytic schizogony to gametocytogenesis is still not understood. Bruce *et al.*[2] showed that merozoites released from a single schizont become either all asexual parasites or all gametocytes, indicating that the switch to sexual differentiation is likely to occur during the preceding asexual red blood cell cycle. Sex determination occurs at the same time or soon after the switch to sexual development, as the entire progeny of a given gametocyte-producing schizont comprises exclusively either male or female cells [3]. The mechanism of parasite commitment to sexual differentiation appears to be constitutive but may be modulated by the environment, as reviewed in [4].

The NIMA-related protein kinases (Neks) constitute a family of eukaryotic serine/threonine kinases implicated in cell cycle control, and whose main role is to regulate centrosome and cilia function [5,6]. The *P. falciparum* kinome includes four Nek kinases, two of which, Pfnek-2 and Pfnek-4, were shown to be predominantly or exclusively expressed in sexual stages, suggesting a possible role in the sexual development of the parasite. Consistent with this hypothesis, it has been previously shown that the rodent malaria parasite *Plasmodium berghei* NIMA-related kinases Pbnek-2 and Pbnek-4, displaying female gametocyte-specific expression, are essential for pre-meiotic genome replication in the zygote and ookinete formation in the mosquito host [7-9]. Here, the expression and function of the *P. falciparum* Nima-related kinase Pfnek-4 were investigated at the onset and during progression of gametocytogenesis. A subpopulation of asexual stage parasites undergoing schizogony and expressing the Pfnek-4 protein was identified. Further analyses indicate that the progeny of these asexual parasites is more likely to differentiate into gametocytes in the subsequent red blood cell cycle. Since not all asexual parasites expressing Pfnek-4 appear to be committed to sexual development, altogether the data suggest that Pfnek-4 identifies a population of committed and reversibly pre-committed parasites.

## Methods

### Molecular cloning and plasmid constructs

Genomic and cDNA sequence data were accessed via PlasmoDB [10]. *The pfnek-4* gene PlasmoDB identifier is PF3D7_0719200, MAL7P1.100 in earlier versions. The Pfnek-4-GFP plasmid (pCHD-Pfnek-4) was generated by using the pHGB and pCHD-1/2 transfection vectors based on Gateway^TM^ recombinational cloning and described in Tonkin *et al.*[11]. The 997-bp 5’-flanking region of Pfnek-4 was amplified from 3D7 genomic DNA using the forward OL-306 (GGGTCGACGAACTCATCATTCATA) and reverse OL-308 (CCAGATCTTGAATGGTTATAAGATATAC) oligonucleotides containing *Sal*I and *Bgl*II sites, respectively. The 933-bp Pfnek-4 open reading frame was amplified from a gametocyte cDNA library using the oligonucleotides forward OL-598 (CCCAGATCTATGAATAAATATGAAAAGATTAGAG) and reverse OL-599 (CCCCCTAGGAGTATCAACAACATCCAG) containing *Bgl*II and *Avr*II sites, respectively. The digested products, ~1-kb 5’-flanking region and open reading frame of Pfnek-4, were sequentially ligated into the plasmid pHGB to produce the pHGB-Pfnek-4-GFP entry clone. This plasmid was used in a recombination reaction with the pCHD-1/2 destination vector containing the cassette responsible for expression of hDHFR conferring resistance to WR99210 treatment, to produce the final transfection vector pCHD-Pfnek-4-GFP.

The pCC1 and pCC4 vectors constructed for negative selection of single crossover recombinants in the generation of knock-out parasites have been described [12] and formed the basis for the generation of plasmids pScCDUP_Pfnek-4_ and phDHFR_Pfnek-4_ described here. The pScCDUP_Pfnek-4_ plasmid was constructed by replacing the 857-bp SacII-XhoI hsp86 5’ sequence of pCC4 by the 997-bp 5’-flanking region of *pfnek-4* amplified from genomic DNA with forward OL-1082 (GGCCGCGGGAACTCATCATTCATA) and reverse OL-1083 (GGCTCGAGTGAATGGTTATAAGATATAC) containing *Sac*II and *Xho*I sites, respectively, generating an expression vector in which the yeast cytosine deaminase gene is placed under the control of the *pfnek-4* promoter. The phDHFR_Pfnek-4_ plasmid was constructed by replacing the ~1.1–kb *Xho*I-*Xma*I yeast cytosine deaminase coding sequence from the pScCDUP_Pfnek-4_ plasmid by ~0.6–kb of the hDHFR sequence amplified from plasmid pCC1 with forward OL-1084 (GGCTCGAGATGCATGGTTCGCTAAACTGC) and reverse OL-1085 (GGCCCGGGTTAATCATTCTTCTCATATAC) containing *Xho*I and *Xma*I sites, respectively, generating an expression vector in which the hDHFR gene is placed under the control of the *pfnek-4* promoter.

A *pfnek-4* gene disruption plasmid was produced in the plasmid pCAM-BSD that contains the gene conferring resistance to blasticidin. The oligonucleotide pair forward OL-46 (GGGGGGATCCAATTATGGAAATACAATACT) and reverse OL-47(GGGGCGCCGGCGTGGACTTAAATAATAAGG) containing *Bam*HI and *NotI* sites was used to amplify a 1,017 bp fragment for insertion to pCAM-BSD. Ring-stage parasites were electroporated with 50–100 μg plasmid DNA, as previously described. Blasticidin (Calbiochem) was added to a final concentration of 2.5 μg/ml 48 hours after transfection to select for transformed parasites. Resistant parasites appeared after three to four weeks and were maintained under selection. After verification by PCR that *pfnek-4*^*-*^ parasites were present, the population was cloned by limiting dilution in 96-well plates (0.25/0.5/1.0 parasite per well). Genotypic analysis enabled selection of independent *pfnek4*^-^ clones for further phenotypic analysis. All constructs were sent to the Dundee Sequencing Service, University of Dundee, UK, for sequence verification before being used.

### Parasite culture, transfection and determination of the growth rate

The 3D7 clone of *P. falciparum*, its F12 subclone and the 3D7 and F12 transfectants were grown in human erythrocytes using 0.5% Albumax II (Invitrogen) and synchronized using sorbitol as described previously [13]. For transfections, synchronized ring-stage parasites (3D7 and F12) were electroporated with 50–100 μg of plasmid DNA using standard procedures. Transformed parasites were selected in presence of 5 nM WR22910 (Jacobus Pharmaceutical Co Inc, Princeton, NJ, USA) or 2.5 μg ml^-1^ blasticidin (Calbiochem). Parasitaemia was monitored by Giemsa-stained thin blood smears. Induction of gametocytogenesis was performed according to the protocol of Carter *et al.*[14], culturing asexual blood stage parasites for four to five days in 6%-haematocrit blood cultures to high 8-10% parasitaemia.

### Nested RT-PCR and diagnostic PCR

Total RNA samples were extracted from parasite pellets using TRIzol lysis solution (Invitrogen). DNase treatment of RNA samples prior to RT-PCR was performed by incubation at 37°C for 30 min using the RQ1 RNase-free DNase I purchased from Promega. The DNase was inactivated by incubation at 65°C for 10 min. RT-PCRs were performed with 500 ng of total RNA/reaction using the ImPromII reverse transcription system purchased from Promega. The RT reactions were incubated at 42°C for 1 h. For the first round of PCR (30 cycles at 94°C for 45 sec, 55°C for 45 sec, and 68°C for 2 min), Pfnek-4-specific primers were forward OL-587 (GAGAGGGATCCATGAATAAATATGAAAAGA) and reverse OL-586 (GAGAGGTCGACTTAAGTATCAACAACATCC). For the second round of PCR (25 cycles at 94°C for 45 sec, 55°C for 45 sec, and 68°C for 2 min), 1 μl (1/25) of each PCR product was reamplified using the Pfnek-4-specific primers forward OL-46 (GGGGGGATCCAATTATGGAAATACAATACT) and reverse OL-47 (GGGGCGCCGGCGTGGACTTAAATAATAAGG).

Disruption of the *pfnek-4* gene was analysed by diagnostic PCR using three primer pairs. The primer pair forward OL-761(CACGACATTACATAATAAAAGC) and reverse OL-763 (ATCCCTTTTATGAATTTACTG) produced a 1288-bp fragment corresponding to the undisrupted *Pfnek-4* locus from wild-type 3D7. Primer pairs forward OL-761 and reverse OL-168 (CAATTAACCCTCACTAAAG), and forward OL-167 (TATTCCTAATCATGTAAATCTTAAA) and reverse OL-764 (TCGAAGAGGTCATTATATATC) amplified across the 5’ and 3’ ends of the integration site, giving rise to 1,179- and 1,277-bp products only in the disrupted locus, respectively.

### Western blot analysis

Western blot analysis was performed on cell-free extracts prepared by resuspending parasite pellets in M-PER Mammalian Protein Extraction Reagent (Pierce) supplemented with 1 mM phenylmethylsulphonyl fluoride and Complex^TM^ mixture protease inhibitor tablet from Roche Applied Science. Immunoblotting was performed as described [13], using monoclonal anti-GFP antibodies (Roche) and horseradish peroxidase-conjugated sheep anti-mouse IgG antiserum (Sigma). Lambda protein phosphatase (New England BioLabs) was used to dephosphorylate protein extracts (20 μg) 30 min at 30°C following the supplier recommendations prior to western blot analysis.

### Fluorescence microscopy

Live imaging of parasite transfectants was performed on a Delta vision deconvolution fluorescence microscope (100x/1.4 oil immersion objective Olympus IX-70). Images were processed using IMARIS version 7.0. For live imaging, parasite nuclei were stained by incubation 10 min at 37°C in complete medium containing 1 μg ml^-1^ Hoechst 3342 (Invitrogen).

### Flow cytometry and cell sorting

Flow cytometer analysis of parasite subpopulations was performed on a FACScan flow cytometer (Becton Dickinson Biosciences). Cells were either fixed in 0.025% glutaraldehyde and propidium iodide (PI)-stained or directly stained with 1 μg ml^-1^ Hoechst 33258 (Invitrogen) prior to analysis. The threshold was set to a value eliminating uninfected red cells. For sorting Pfnek-4-GFP-expressing transfectants, late trophozoite-stage parasites were first enriched by using the VarioMACS separator and CS MACS columns (MiltenyiBiotec), then returned to culture conditions for three to four hours prior to cell sorting using a FACS ARIA II SORP Instrument (Becton Dickinson Biosciences). In two independent experiments, Pfnek-4-GFP^-^ (4 10^6^ and 12 10^6^) and Pfnek-4-GFP^+^ (0.8 10^6^ and 1.6 10^6^) parasites, respectively, were collected over a four-hour cell sorting period and returned to culture conditions in flat-bottom 96-well plates with fresh red blood cells to 2% -haematocrit and 1% -parasitaemia. Gating of GFP^-^ and GFP^+^ parasite populations has been set in a restrictive way such that the sorted cells exhibited high levels of purity in both experiments (purity of GFP^-^ sorted cells, 100%; GFP^+^ sorted cells, 92.8 and 97.2%) when re-analysed by FACS analysis.

## Results

### Transgenic expression of a Pfnek-4-GFP fusion protein in gametocytes and a subset of asexual blood stage parasites

**Figure 1:**
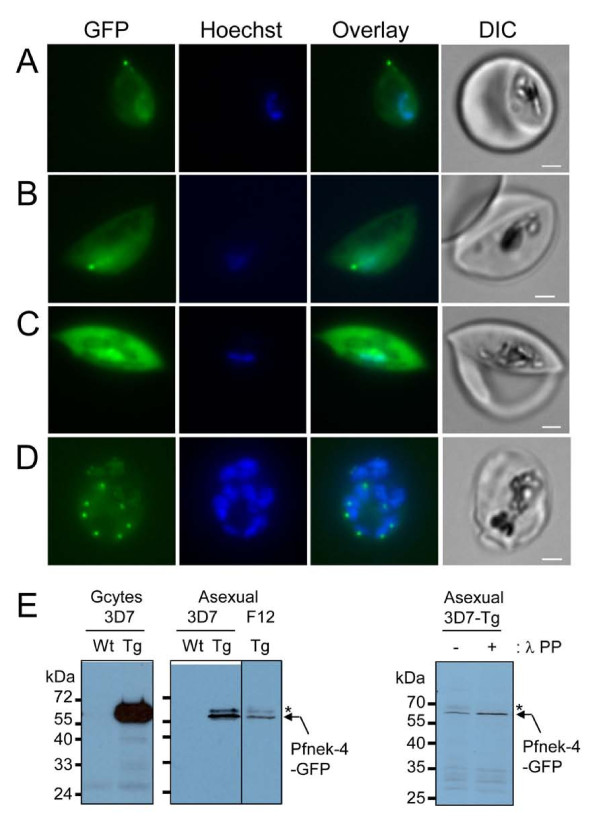
**Episomal expression of GFP-tagged Pfnek-4 protein in gametocytes and a subset of asexual stage parasites.** Live images of Hoechst-stained stage II (**A-B**), stage III gametocytes (**C**), and multinucleated-schizont stage (**D**), from transgenic 3D7 parasites expressing GFP-tagged Pfnek-4 controlled by cognate (Pfnek-4) promoter. Overlay between the green and blue channels, and corresponding DIC images are shown as well. Scale bars, 2.0 μm. **E**, Immunoblots showing ~62-kDa protein band (arrow) recognized by mouse anti-GFP mAbs (Roche) using parasite extracts (10 μg) from wild type (Wt) 3D7 and Pfnek-4-GFP transgenic (Tg) stage II-V gametocytes and synchronized schizonts of 3D7 and the non-gametocyte-producer F12 clones at day 4 of induction of gametocytogenesis, and from Pfnek-4-GFP transgenic 3D7 synchronized schizonts before (−) and after (+) 30 min-incubation with λ-protein phosphatase. High MW Pfnek-4-GFP species susceptible to λ-protein phosphatase treatment are marked with an asterisk. Note the much lower expression level in asexual parasite extracts as compared to gametocyte extract.

*P. falciparum* 3D7 parasites were transfected with a plasmid containing the Pfnek-4 coding sequence fused C-terminally to GFP under the control of its cognate (Pfnek-4) promoter, using ~1 kb of genomic sequence upstream of the Pfnek-4 translation initiation codon. Episomal propagation of the construct was maintained in the presence of antifolate drug pressure. Stage II transgenic gametocytes displayed a single punctuate Pfnek-4-GFP fluorescent structure, which coincides with or is located nearby one tip of the parasite and which is often closely associated with the nucleus (Figure 1A-B). The Pfnek-4-GFP protein appeared to accumulate in the cytosol of stage III gametocytes (Figure 1C) and remained present at high levels throughout the formation of mature stage V gametocytes (Additional file 1, panel A). Interestingly, while the bulk of examined schizonts did not show any detectable signal, as expected for a protein thought to be gametocyte-specific, fluorescence microscopy of asexual stage cultures consistently identified a small subpopulation (a few percents, see below) of schizonts with dots (single or doublets) of concentrated Pfnek-4-GFP protein associated with nuclei (Figure 1D). Fluorescence was never detected in ring and trophozoite stages. Noteworthy, all nuclei within a single schizont appeared to be associated with punctuate Pfnek-4-GFP fluorescence from early developing schizont to multinucleated schizonts (Additional file 1, panel B).

Western blot analysis of parasite extracts from gametocyte and asexual blood stage parasite cultures, using anti-GFP antibodies, detected a specific protein of ~62 kDa, consistent with the predicted mass of the Pfnek-4-GFP fusion protein (Figure 1E). This is in contrast to the previously reported lack of detectable Pfnek-4 protein in cultures of asexual 3D7 parasites, using chicken anti-Pfnek-4 IgY antibodies. Possible explanations for this discrepancy include: (i) the use of synchronized schizont stage parasites grown under conditions inducing gametocyte formation in the present series of experiments (in contrast to mixed asexual not under gametocytogenesis-inducing conditions in the earlier study [8]), and (ii) higher sensitivity of the anti-GFP monoclonal antibodies *versus* the anti-Pfnek-4 IgY. Pfnek-4-GFP transfectants produced in the F12 clone background, a 3D7 subclone having lost the ability to produce gametocytes, allow to exclude that the Pfnek-4 signal from 3D7 asexual cultures is solely caused by contaminating gametocytes (Figure 1E). Anti-GFP reactive protein species of slightly higher molecular mass likely corresponds to Pfnek-4 phosphorylated forms as indicated by their susceptibility to λ protein phosphatase treatment (Figure 1E).

**Figure 2:**
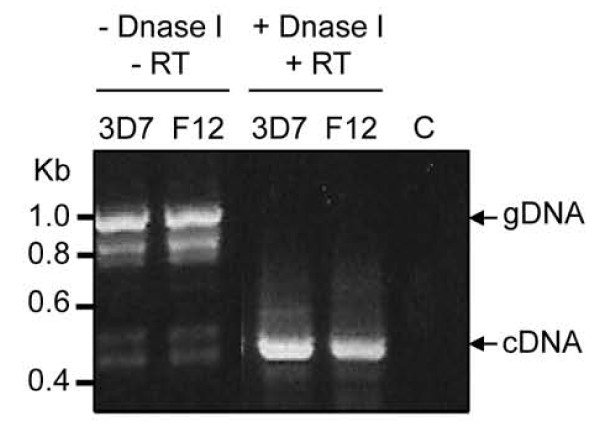
**Expression of Pfnek-4 in untransformed asexual parasites.** Nested RT-PCR detection of Pfnek-4 transcripts in untransformed gametocyte-less *Plasmodium falciparum* F12 substrain. Two rounds of amplification performed on RNA samples untreated or treated with Dnase I and reverse transcriptase from both 3D7 and F12 clones yielded amplified fragments of the expected size for Pfnek-4 genomic DNA (0.99 kb) and cDNA (0.48 kb). C, water control.

Nested RT-PCR using RNA isolated from asexual stages of the 3D7 parental clone and the gametocyte-less F12 clone yielded amplicons of the expected size for Pfnek-4 cDNA (Figure 2), the identity of which was verified by cloning and sequencing, further demonstrating that Pfnek-4 is not strictly gametocyte-specific, and that low mRNA levels are also present in untransformed asexual parasites.

### Asexual stage parasites expressing Pfnek-4 display high rate commitment to sexual differentiation

**Figure 3:**
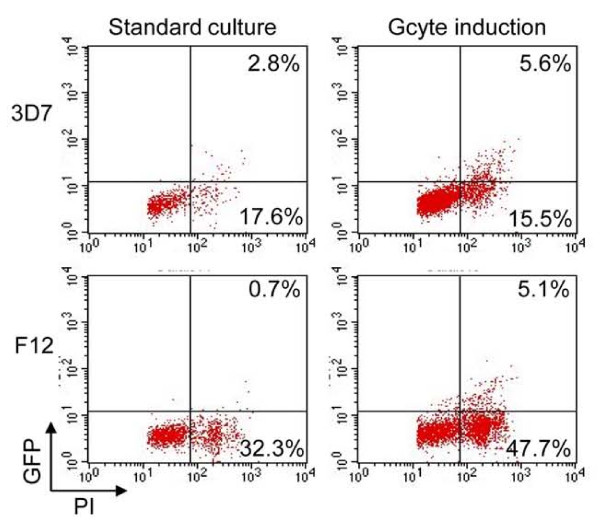
**Culture conditions inducing sexual differentiation result in increased number of multinucleated-schizont stage parasites expressing Pfnek-4.** Dot plots representations of GFP and propidium iodide (PI) fluorescence intensity of Pfnek-4-GFP 3D7 (upper panels) and F12 (lower panels) transfectants grown for four days either in standard culture conditions (low parasitaemia and every second-day dilution) or in conditions known to induce commitment to gametocytogenesis (high haematocrit blood cultures and high parasitaemia). Cells were fixed with 0.025% glutaraldehyde, stained for DNA with the fluorescent dye propidium iodide (PI) and subsequently analysed by a FACScan flow cytometer (Becton Dickinson). Uninfected red blood cells with low FL-2 (PI) fluorescence were excluded from the selection. Parasite numbers are given related to total number of infected red cells.

Pfnek-4-GFP transgenic 3D7 and F12 parasites, either maintained under standard culture conditions or grown under conditions inducing commitment to sexual differentiation were subjected to FACS analysis. Figure 3 shows dot plots of samples of glutaraldehyde-fixed, propidium iodide (PI)-stained 3D7 and F12 transfectants grown under standard culture conditions, the GFP-positive (GFP^+^) schizonts consisting of 13.7% and 2.1% of the total schizont population, respectively; the high DNA content as measured by PI fluorescence allow to exclude that these are gametocytes. Under culture conditions stimulating sexual differentiation, 3D7 and F12 GFP^+^ schizonts significantly increased to 26.5% and 9.6% of the total schizont population, respectively. FACS analysis of live, 3D7 Hoechst-stained Pfnek-4-GFP transfectants confirmed the ~2-fold increase (paired *t*-test; p<0.0001) in the asexual multinucleated GFP^+^ parasite population in culture conditions inducing sexual differentiation (Table 1). It should be stressed that the GFP^+^ asexual parasites generated at high parasitaemia display a punctuate pattern and intensity of Pfnek-4-GFP fluorescence similar to the GFP^+^ asexual parasites observed in standard culture conditions, arguing against any major deregulation of the episomal promoter due to stress conditions (data not shown).

**Table 1:** Flow cytometry analysis of live Pfnek-4-GFP transfectants grown in standard or culture conditions inducing commitment to sexual differentiation

		Multinucleated parasites*	
	**Culture conditions**	**GFP positive (%)**	**GFP negative (%)**
Exp. 1	Standard	5.8 ± 0.4	59.4 ± 0.5
	Day-5 Gcyte induction	11.3 ± 0.8**	57.0 ± 0.5
Exp. 2	Standard	4.6 ± 0.3	48.3 ± 1.9
	Day-5 Gcyte induction	9.3 ± 0.8**	46.3 ± 2.9

*Set as the parasite population with Hoechst fluorescence intensity > 2–3 fold higher than that of the ring-stage (1n) parasite population; values are mean ± SD calculated from three separate cultures obtained in two independent experiments at day-5 of culture.

**Paired *t*-test: two-tailed *p* value < 0.0001.

**Figure 4:**
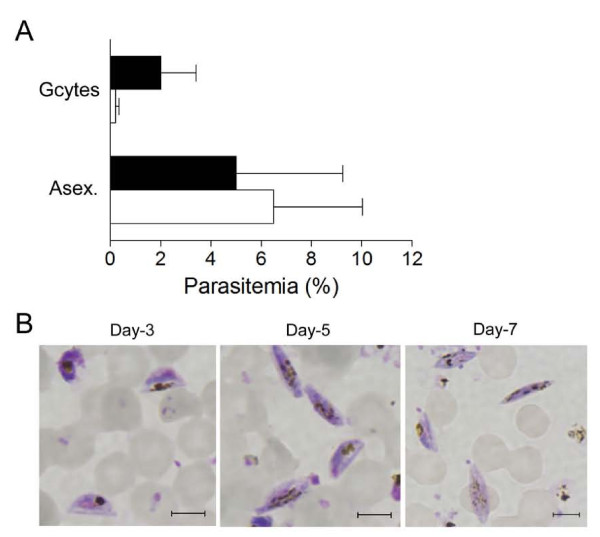
**Commitment of Pfnek-4-GFP**^**+**^**-sorted parasites to sexual differentiation.** Pfnek-4-GFP parasite transfectants grown for four days under conditions inducing commitment to sexual differentiation, were enriched for schizont stages, separated into GFP^-^ and GFP^+^ populations, and returned to *in vitro* culture in presence of fresh red blood cells at ~1% parasitaemia. **A**, Day-4 gametocytes and asexual blood stages parasitaemia (mean ± SD) of GFP^-^ (open bars) and GFP^+^ (closed bars) -sorted parasite cultures as determined by Giemsa-staining of thin smears. **B**, Giemsa-staining of thin smears of sorted GFP^+^-parasites at day 3, 5 and 7 of *in vitro* tissue culture showing the presence of stage II (left panel), III (middle) and IV (right panel) gametocytes, respectively. Scale bars, 5.0 μm.

A cell-sorter approach was used to isolate live GFP-negative (GFP^-^) and GFP-positive (GFP^+^) parasites from the same schizont population and examine the production of gametocytes when returned to *in vitro* tissue culture. Two independent experiments revealed that at day-4 after the FACS-sorted parasites were re-cultivated, the number of gametocytes observed by Giemsa-staining was about 10 times higher in GFP ^+^ −sorted cells (2.2 ± 1.2%) than in the GFP^-^-sorted cells (0.2 ± 0.1%) (Figure 4A). During the same four-day assay -period, asexual parasites increased to 5.0 ± 3.0% parasitaemia for the GFP^+^-sorted cells, and 6.5 ± 2.5% for GFP^-^-sorted cells, respectively. At day 3, 5 and 7 of culture, gametocytes mainly displayed the D shape, elongated shape and pointed ends that characterize stage II, III and IV, respectively (Figure 4B), consistent with development from sorted asexual schizont-stage parasites, rather than commitment following the sorting procedure or the sorting of gametocytes present in the asexual stage population. FACS analysis of Hoechst-stained parasite cultures undergoing sorting in experiments I and II, indicated that early stage gametocytes, *i.e.* GFP^+^ parasites with low DNA content, might account for approximately 5.3 and 12.9% of the sorted GFP^+^ cells, respectively.

### Reporter-gene negative/positive selection of asexual stage parasites expressing Pfnek-4

**Figure 5:**
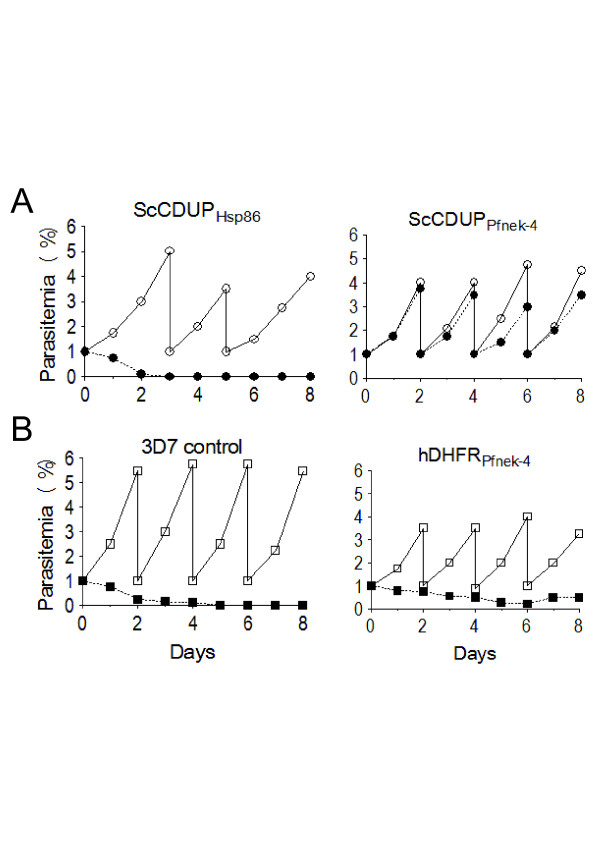
**Selection of parasite transfectants expressing negative or positive selectable markers directed by Pfnek-4 promoter.A**, *In vitro* growth rate of 3D7 parasite transfectants expressing the *Saccharomyces cerevisiae* cytosine deaminase-uridylphosphoribosyltransferase fusion gene (ScCDUP) driven by Hsp86 (pScCDUP_Hsp86_) or Pfnek-4 (pScCDUP_Pfnek-4_) 5’ flanking regions, in presence (●) or absence (○) of prodrug 5-FC (1 μM). **B**, *In vitro* growth rate of untransformed 3D7 (3D7 control) and 3D7 parasite transfectants expressing the human dihydrofolate reductase (hDHFR) gene driven by Pfnek-4 5’ flanking region (phDHFR_Pfnek-4_), in presence (■) or absence (□) of WR22910 (5 nM). Parasitaemia (mean of duplicate cultures) were determined daily by Giemsa-stained blood smears and parasite cultures >3% parasitaemia were diluted to 1%-parasitaemia.

Expression of the gametocyte-specific *pfnek-4* gene in a subpopulation of asexual stage parasites was further investigated using a selection system containing an expression cassette for the bifunctional yeast protein cytosine deaminase/uridyl phosphoribosyl transferase (ScCDUP), providing efficient negative selection of *Plasmodium* upon treatment with the prodrug 5-fluorocytosine (5-FC) [12,15]. Transformed 3D7 parasites expressing the ScCDUP gene under control of the constitutive Hsp86 promoter (pScCDUP_Hsp86_) were rapidly lost in the presence of 5-FC, as expected (Figure 5A). In contrast, transformed 3D7 parasites expressing ScCDUP driven by ~1-kb Pfnek-4 5’ flanking region (pScCDUP_Pfnek-4_) showed a marginal reduction in growth rate in presence of 5-FC, consistent with the Pfnek-4 being active in only a small sub-population of asexual parasites. In parallel, to achieve a positive selection of parasites expressing Pfnek-4, the expression cassette was modified such that the human *dihydrofolate reductase* (hDHFR) was placed under control of ~1-kb 5’ flanking region from Pfnek-4 (phDHFR_Pfnek-4_). Figure 5B shows that positive selection of transformed 3D7 parasites expressing hDHFR driven by the Pfnek-4 promoter with the antifolate drug WR99210 caused a marked reduction in parasitaemia growth rate, but was not sufficient to completely abolish proliferation. In contrast parental 3D7 parasites were fully susceptible, as expected.

### Generation of *Plasmodium falciparum* parasites with a disrupted *Pfnek-4* gene

**Figure 6:**
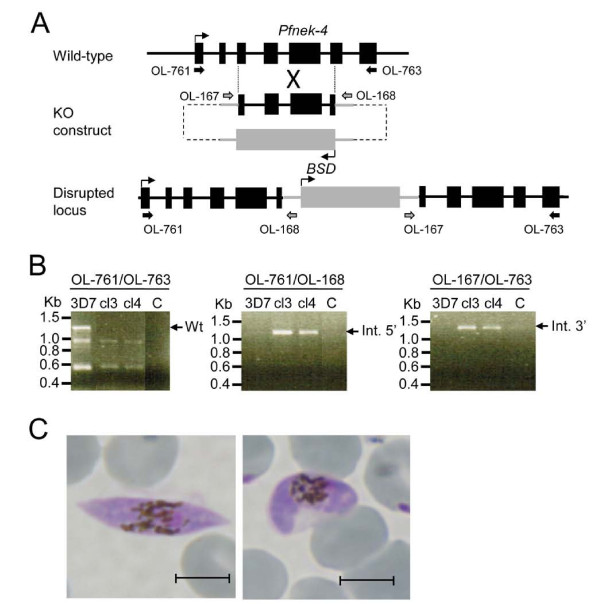
**Disruption of the*Pfnek-4*gene.A**, Strategy for gene disruption. The schematic representation shows the Pfnek-4 locus, the gene-targeting construct used for gene disruption by homologous recombination, and the pseudo-diploid locus resulting from integration of the KO construct. The primers used for the diagnostic PCRs are indicated. BSD, blasticidin-resistance cassette. **B,** PCR analysis indicating disruption of the *Pfnek-4* gene in parasite clones cl3 and cl4 obtained from two independent transfections. Genomic DNA isolated from 3D7 wild-type parasite and *Pfnek-4*^*-*^ clones cl3 and cl4, were subjected to PCR using the indicated primers OL-761 and OL-763 (diagnostic for the wild-type locus), OL-761 and OL-168 (diagnostic for 5’ integration), and OL-167 and OL-763 (diagnostic for 3’ integration). PCR products corresponding to expected sizes for wild-type (1288-bp), 5’ integration (1179-bp) and 3’ integration (1277-bp) are indicated on the right. **C**, Giemsa-staining of thin smears of *pfnek-4*^*-*^ gametocytes at days 12–14 of *in vitro* tissue culture stimulating gametocyte production, showing the presence of stage IV (left panel) and stage V (right panel) gametocytes. Scale bars, 5.0 μm.

The function of the *nek-4* gene has been previously investigated in the rodent malaria parasite *Plasmodium berghei*; it was shown that disruption of *Pbnek-4* does not affect asexual replication, gametocytogenesis and gametogenesis, but impairs differentiation of zygotes into ookinetes [8]. To investigate a possible phenotype caused by the absence of a functional Nek-4 enzyme in the commitment of *P. falciparum* parasite to sexual differentiation, the *pfnek-4* gene of the 3D7 parasite line was disrupted by single-crossover homologous recombination (Figure 6A). Clonal lines derived from two independent transfection experiments were established by limiting dilution, and their genotypes were analysed by PCR. The amplicon corresponding to the wild-type locus was not detected in clones cl3 and cl4, but was observed in untransfected 3D7 parasites. In contrast, amplicons diagnostic for the 5’ and 3’ boundaries of plasmid integration were detectable in the transgenic parasites (Figure 6B). The generation of *pfnek-4*^*-*^ mutant parasite clones demonstrates that the gene is not essential for replication in erythrocytes. Moreover, no effect on parasite growth rate of the *pfnek-4*^*-*^ mutant parasites was observed (data not shown). At day 12 to 14 of culture the number of gametocytes (~0.5-0.7%) appears undistinguishable from those of 3D7 wild-type parasite cultures and the majority of gametocytes were at stage IV and V as assessed by Giemsa-stained smears (Figure 6C). Most stage V gametocytes displayed the morphological characteristics of mature female gametocytes [16], indicating that *pfnek-4* disruption does not affect the formation of mature female gametocytes.

## Discussion

In *P. falciparum*, the commitment to gametocytogenesis occurs during the preceding asexual RBC cycle [2]. The present study identifies a subset of schizont-stage parasites expressing Pfnek-4, a protein kinase previously thought to be gametocyte-specific. Upon sorting, Pfnek-4-GFP positive schizonts produced elevated levels of gametocytes in the subsequent red blood cell cycle. The experimental approach is based on episomal expression of Pfnek-4-GFP fusion protein driven by 1 kb-genomic DNA upstream of the Pfnek-4 coding sequence. It cannot be formally excluded that the proper expression pattern is not maintained in this conditions. However, a study by Khan *et al.*[7] reported the ability of a 678 bp-*Pbnek-4* promoter to drive the expression of a reporter GFP protein in female gametocytes, but not in male gametocytes or asexual blood stages. In this latter study, nine out of ten promoters (the only exception being most likely due to the short 5’ region available from the genome database), were found to drive the expression of the GFP reporter protein in agreement with the prediction from proteomic analyses, suggesting that sex-specific expression is mainly controlled by the 5’ UTR/promoter. In another instance, the episomal expression of the HA-epitope-tagged *P. falciparum* protease, PfROM1, placed under control of its own promoter element, revealed an expression at mature stages and localization to the mononeme, a newly described apical organelle of *P. falciparum* merozoites [17]. The nucleus-associated punctuate pattern observed for Pfnek-4-GFP distribution, which is reminiscent of what is observed with the *P. falciparum* Aurora-related kinase Pfark-1, is consistent with the location of Nima- and Aurora-related kinases, many members of which associate with centrosomal structures (see below). Thus, it is proposed that there is a high likelihood that the localization observed with the episomally-encoded Pfnek-4-GFP fusion protein might reflect that of the endogenous enzyme.

It should be stressed that: (i) the sorted Pfnek-4-GFP positive schizont population not only generated gametocytes but also parasites undergoing asexual RBC cycles; and (ii) a proportion of the parasites positively selected for expression of the hDHFR resistance marker driven by the *pfnek-4* gene was still able to undergo asexual cycles. Altogether these findings support and expand previous studies indicating that malaria parasites have quantitative sensitivity to gametocyte induction and that multiple stimuli can induce gametocytogenesis, reflecting the highly flexible mechanism underlying sexual differentiation [4]. Noteworthy, high rate conversion to sexual forms of asexual parasites grown at high densities was shown to be reversible by dilution within a time window smaller than the time required for one asexual cycle (48 hours) [2]. This feature might explain the relatively lower gametocyte conversion rate observed in the GFP^+^ sorted parasites (2.2%), as compared to normal 3D7 parasites grown under gametocyte-inducing culture conditions (~4-5%). Altogether, the data suggest that Pfnek-4 identifies a population of committed and reversibly pre-committed parasites. Since sex determination appears to occur simultaneously to commitment to sexual differentiation, the asexual subpopulation expressing Nek-4, a protein shown to be restricted to female gametocytes in the rodent malaria parasite *P. berghei*[7,8], is likely to represent the progenitor of female rather than male gametocytes, although this question remains to be further investigated.

The expression of a gametocyte-specific gene product in sexually-committed asexual parasites is consistent with a developmental change in gene expression during sexual differentiation [18,19], and extends previous studies reporting the expression of gametocyte-specific genes, such as Pfs16, in sub-populations of asexual-stage parasites [9,20,21]. Since some of the asexual parasite population expressing Pfnek-4 did not appear to be fully committed to sexual differentiation (being still able to undergo RBC cycles, see above), a switch to gametocyte-specific gene expression may occur before the “no-return”decision to commit to gametocytogenesis is made. Analysing genes expressed in the sexually-committed population would be of great interest to explore gene regulation in the context of commitment to gametocytogenesis, and would help to identify signatures of early sexual development of malaria parasites. Identification of Pfnek-4 as a molecular marker of sexually-committed schizonts provides a useful tool, making this parasite population amenable to purification followed by transcriptome and proteome analyses. Transcriptional regulator candidates controlling expression of subsets of genes are the ApiAP2 family of proteins. De Silva *et al.*[22], reported highly coherent expression patterns of predicted downstream targets of *P. falciparum* AP2 transcription factors, suggesting essential roles in parasite development. Translational regulation also plays a critical role during commitment to gametocytogenesis [18,19]. Targeted disruption of PfPuf2, a member of the Puf family of translational repressors was shown to promote the formation of gametocytes and the differentiation of male gametocytes [23].

In *P. berghei*, the Nek-4 kinase does not appear to be required for gametocytogenesis but is essential for pre-meiotic DNA replication in the zygote, consistent with cell-cycle related functions [7,8]. That *pfnek-4*^*-*^*P. falciparum* parasites are able to undergo gametocytogenesis and produce mature stage V gametocytes, indicates that Pfnek-4 is not required for the early stages of the sexual cycle in both *P. berghei* and *P. falciparum*. This conclusion is also supported by the finding that *pfnek-4*^*-*^ clones produce female gametocytes. It is intriguing that the timing of recruitment of the Pfnek-4 protein to schizont nuclear bodies appears to coincide with the occurrence of nuclear divisions. Noteworthy, all nuclei within a single schizont appear to be associated with punctuate Pfnek-4-GFP fluorescence from early developing to multinucleated schizont, in contrast to Pfark-1, a mitotic kinase that marks only a subset of nuclei in a given schizont as a result of transient recruitment at the spindle pole bodies, a consequence of asynchronous nuclear division in a single schizont [13]. In contrast to Pfark-1, the Pfnek-4-GFP protein appears to associate to all nuclei, irrespective of their nuclear division status. Furthermore, cell cycle-arrested stage II gametocytes were found to express the Pfnek-4-GFP protein with a punctuate fluorescence similar to parasites undergoing schizogony. Preliminary results showing a close association of doublets of Pfnek-4-GFP fluorescence with short mitotic spindle microtubules in schizont-stage parasites (data not shown), are consistent with a recruitment of Pfnek-4 at nuclear spindle pole bodies, the centrosome equivalent of *Plasmodium* parasites, an observation consistent with the known centrosomal functions of Nima-related kinases. However, the sub-cellular structure to which Pfnek-4 associates remains to be better defined. Whether the Pfnek-4 enzyme has cell-cycle-related functions in RBC-stages still remains to be elucidated.

## Conclusions

Taken together, the data presented in this study indicate that the Nima-related kinase Pfnek-4 identifies a small subset of schizont-stage *P. falciparum* parasites displaying high rate conversion to sexual differentiation, and thus represents a molecular marker of sexually-committed and -pre-committed schizonts. Pfnek-4 does not appear to control the switching of asexual stages into gametocytes.

## Competing interests

The authors declare that they have no competing interests.

## Authors’ contributions

LR carried out molecular cloning, RT-PCR, western blotting, parasite genetic manipulations, analyses by fluorescence microscopy and flow cytometry, and participated in conception of the study and writing of the manuscript. MG performed the cell sorting. AT performed and analysed parasite growth. SM performed and analysed parasite growth and participated in writing of the manuscript. CD participated in conception of the study and writing of the manuscript. All authors read and approved the final manuscript.

## Supplementary Material

Additional file 1 **Expression of the Pfnek-4-GFP protein in gametocytes, early and multinucleated schizont-stage 3D7 transfectants.** Live cell images of stage III, IV and V gametocytes (A), early developing (2–3 nuclei stage) (higher panel) and multinucleated (lower panel) schizonts (B) from 3D7 transfectants stained with Hoechst 33258. The Pfnek-4-GFP protein strongly accumulates in the cytosol of stage III to V gametocytes. Images of early developing schizont and multinucleated schizonts show that each of Hoechst-stained nuclear bodies is associated with a dot of Pfnek-4-GFP fluorescence (green). Overlay of all channels (A, B), and corresponding DIC images are shown as well (B). All images were acquired using a Deltavision RT wide-field epifluorescence microscope imaging system and a 100x/1.4 objective and processed using SoftWorx software. Image analysis software was IMARIS version 5.0. Scale bars, 2.0 μm. Click here for file
