# Supplementary figures and images for: The Plasmodium falciparum, Nima-related kinase Pfnek-4: a marker for asexual parasites committed to sexual differentiation
Source: Malar J. 2012 Jul 31;11:250. doi: 10.1186/1475-2875-11-250 (PMC3495404; doi:10.1186/1475-2875-11-250)

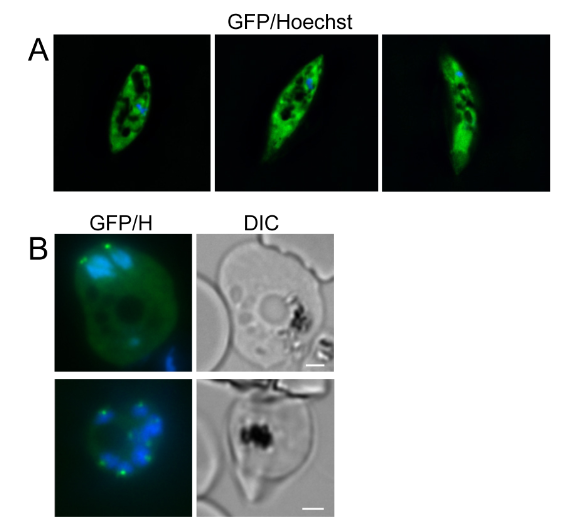

Supplement: Additional file 1 — Expression of the Pfnek-4-GFP protein in gametocytes, early and multinucleated schizont-stage 3D7 transfectants. Live cell images of stage III, IV and V gametocytes (A), early developing (2–3 nuclei stage) (higher panel) and multinucleated (lower panel) schizonts (B) from 3D7 transfectants stained with Hoechst 33258. The Pfnek-4-GFP protein strongly accumulates in the cytosol of stage III to V gametocytes. Images of early developing schizont and multinucleated schizonts show that each of Hoechst-stained nuclear bodies is associated with a dot of Pfnek-4-GFP fluorescence (green). Overlay of all channels (A, B), and corresponding DIC images are shown as well (B). All images were acquired using a Deltavision RT wide-field epifluorescence microscope imaging system and a 100x/1.4 objective and processed using SoftWorx software. Image analysis software was IMARIS version 5.0. Scale bars, 2.0 μm. [file 1475-2875-11-250-S1.png]
